# Conservative Treatment of an Invaginated Maxillary Lateral Incisor with a C-shaped Canal Using Cone-Beam Computed Tomography

**DOI:** 10.7508/iej.2015.04.014

**Published:** 2015

**Authors:** Maryam Forghani, Elaheh Moghim Farooji, Javad Abuchenari, Maryam Bidar, Neda Eslami

**Affiliations:** a *Dental Materials Research Center, Department of Endodontics, Dental School, Mashhad University of Medical Sciences, Mashhad, Iran*; b * Endodontist, Mashhad, Iran;*; c * Dental Research Center, Department of Endodontics, Dental School, Mashhad University of Medical Sciences, Mashhad, Iran;*; d *Dental Research Center, Department of Orthodontics, Dental School, Mashhad University of Medical Sciences, Mashhad, Iran*

**Keywords:** C-Shaped Canal, Cone-Beam Computed Tomography, Dens Invagination, Dens Invaginatus, Dens in Dente, Invaginated Teeth, Maxillary Lateral Incisor

## Abstract

This report describes the non-surgical treatment of an invaginated maxillary lateral incisor with two fused roots. The mesial root had a C-shaped canal, while the distal one had a *type III* dens invagination. Cone-beam computed tomography (CBCT) was used to help with the diagnosis and treatment decision making. Clinical and radiographic follow-up revealed satisfactory periapical repair and absence of symptoms after 15 months.

## Introduction

A dequate information about the anatomy of the pulp and its variations is necessary for an efficient endodontic treatment [1]. Tooth abnormalities are caused by genetic disorders or environmental factors during tooth formation [2]. These abnormalities including variations in the shape of the crown and the number/shape of the roots, are common in maxillary lateral incisors. In the literature, 6.6% of the maxillary lateral incisors are reported to have more than 2 roots [3, 4].

Shape and number of the roots are determined by epithelial root sheath during maturation of the tooth organ. Failure in connection of this sheath through the lingual or buccal surface can cause the formation of C-shaped roots and canals. Also, C-shaped roots may be created by coalescence due to cement deposition through the time [3]. The frequency of C-shaped canals varies from 2.7% to 44.5% in mandibular second molars in different populations [5, 6]. This anatomic variation can be found in mandibular first and third molars, followed by maxillary molars and first premolars and finally by the maxillary laterals incisors [3].

Dens invagination (*aka* dens in dente) is another developmental abnormality with 0.25-10% frequency, which is common in maxillary lateral incisors [7]. This abnormality is caused by invagination of the enamel organ before its calcification [2, 8, 9]. Oehlers [10] divides this abnormality into three types according to its severity. In the first type, the invagination is restricted to the crown. In the second type, the invagination passes the cemento-enamel junction (CEJ) and in the third type, invagination extends CEJ and can have a separate foramen. 

Presence of these developmental abnormalities can alter the endodontic treatment and its prognosis [11]. Therefore, it is necessary to diagnose their presence and evaluate the treatment intensity before the start. This manuscript reports the endodontic treatment of an invaginated maxillary left lateral incisor with periapical lesion and 3 roots that had a C-shaped anatomy in the distal root canal.

## Case Report

An 18-year old female was referred by a general dental practitioner to the Department of Endodontics of Mashhad University of Medical sciences, Mashhad, Iran. The patient complained of pain and swelling, in the left maxillary anterior segment.

**Figure 1:**
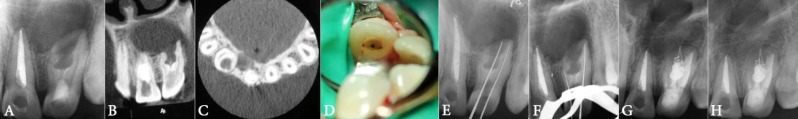
*A)* Initial radiography, *B)* Pre-operative coronal slice of the CBCT image of the maxillary left lateral incisor, *C)* Axial slice,

*D)* Image of three orifices, *E)* Working length determination, *F)* Master apical cone placement, *G) *Immediate post-operative radiograph,

*H)* Follow-up radiography 15 months later

On clinical inspection a localized swelling in the gingiva of the left lateral incisor was evident and the tooth had a temporary filling. The maxillary left lateral incisor was not responsive to electronic pulp testing (Parkell Electronics*. *Division*,* Farmingdale, NY, USA) and pulp vitality tests with heat and cold. Patient reported discomfort on percussion as well as pain on palpation of periapical area. The tooth presented normal size and color and there was no sinus tract.

The periapical radiographic examination revealed an invaginated lateral incisor with 3 roots and a large periapical radiolucency (Figure 1A). According to the clinical and radiographic examination, the diagnosis was pulp necrosis with an abscess developed within an existing chronic apical periodontitis. Thus conventional root canal therapy was indicated. A cone-beam computed tomography (CBCT) scan was indicated to observe the three-dimensional image of this complex anatomy. The CBCT image taken with Planmeca Promax 3D (Planmeca, Helsinki, Finland) device showed 3 roots: distal root with a C-shaped canal, mesial root with *type III* dens invagination and a middle root with a single canal (Figures1B and C).

The tooth was anesthetized using buccal infiltration of 2% lidocaine containing 1:80000 epinephrine (Darupakhsh, Tehran, Iran) and then isolated. The temporary restoration was removed and the central canal was exposed. After mesiodistal extension of the coronal access, orifices of the two other canals was detected (Figure 1D).

The Ribbon-shaped orifice of the distal canal started at the palatal aspect of the tooth, and then swept around the distal side to end at the distobuccal angle. The invaginated mesial root canal was calcified and could be negotiated with #10 file (Dentsply Maillefer, Ballaigues, Switzerland) to the possible length. The central canal was wide, conical and straight, with pus drainage. After determination of working length (Figure 1E), all 3 canals were instrumented using the step-back technique and irrigated with 5.25% sodium hypochlorite (NaOCl) during the instrumentation. After biomechanical preparation of root canals, calcium hydroxide paste (Golchadent, Karaj, Iran) was mixed with saline and inserted into the canal; the access cavity was temporarily sealed with Cavit )Ariadent, Coltosol, Tehran, Iran).

In the next appointment after 14 days, the patient had no pain and swelling. After rubber dam isolation, calcium hydroxide was removed with master apical file and NaOCl irrigation. Working length of mesial canal was 5 mm and the canal was filled by mineral trioxide aggregate (MTA, Dr Lotfi, Tabriz, Iran). The C-shaped distal canal was filled with #35 gutta-percha (Ariadent, Tehran, Iran) using a lateral condensation technique and AH-26 (Dentsply, Tulsa Dental, Tulsa, OK, USA) root canal sealer. In the central canal which was wide and irregular in coronal and middle zones, the apical third was filled with lateral condensation of #30 gutta-percha and the middle and coronal regions were filled using injection-molded thermoplasticized gutta-percha (Beefill device, VDW, Munich, Germany) delivery system (Figures 1F and G). One week after completion of endodontic treatment, patient had no clinical symptom and the tooth was restored with composite resin (Gradia Direct, GC Corporation, Tokyo, Japan). After15 months of follow-up, the patient had no symptoms and the radiography showed healing of periapical lesion (Figure 1H).

## Discussion

This report represented the endodontic treatment of an invaginated maxillary left lateral incisor with periapical lesion and 3 roots that had a C-shaped anatomy in the distal root canal. Clinical and radiographic follow-up revealed satisfactory periapical repair and absence of symptoms after 15 months. 

Maxillary incisors and more specifically the lateral incisors are often affected by abnormalities such as talon cusp, dens invagination and palatogingival groove. It is assumed that the small tooth organ of these teeth can be influenced by the pressure of larger neighboring tooth buds of central incisor and canine that have been developed several months earlier [12, 13]. The created pressure on tooth germ during morpho differentiation may cause buckling (outfolding or infolding) of the dental lamina [14]. It seems that these malformations are commonly related to genetic disorders because they have ethnical tendency and are more routinely found in specific ethnic groups; moreover they are often accompanied with other anomalies. According to Moyer *et al. *[15], the most distal tooth in each group shows the most diversity in size, shape and calcification time. However, coincidence of several tooth abnormalities is uncommon.

It has been reported that the maxillary lateral incisors are the most common teeth with these abnormalities (85%) with the 3.3% frequency of type *III* invagination [16]. In this type, the invagination penetrates into the apical region throughout the root which develops a second foramen in apical or periodontal region, without the pulp being directly involved [17, 18]. Invagination helps the external irritants reach the isolated pulp which is covered by a thin layer of enamel/dentin or both. In some cases the enamel layer is incomplete. Also there may be a patent canal between the invagination and the pulp [19]. Alani and Bishop [19] suggest that if periodontitis is present around the invaginated tooth with a healthy pulp, all the effort must be made to maintain pulp vitality. In the present case, endodontic treatment of the main canal was conducted because of pulp necrosis and presence of pus.

Conventional radiography provides a two-dimensional image of a three-dimensional structure [20]. Using CBCT and dental operating microscope (DOM), enables recognition and decision making in non-surgical treatments in most complicated cases of dens invagination [10]. In the present case, accurate morphology of the root canal was diagnosed with the help of CBCT.

For the present case, for the root canal filling of the mesial canal, MTA was applied because of its biological compatibility, its healing power by induction of mineralized tissue deposition, excellent sealing power in wet condition and also declined treatment time [18].

Diagnosis of dens in dente is usually accidental. An uncommonly large, peg or barrel-shaped crown or a deep foramen caecum may clinically represent dens invagination [12, 21].

The existence of C-shaped canal in the invaginated teeth has already been reported [22]. It is still a matter of debate whether this type of canal is most routinely seen in invaginated teeth or not. The response to this question is especially of utmost importance in the prognosis of treating the invaginated teeth. Using microscope and CBCT are helpful in the correct diagnosis and treatment of the invaginated teeth.

## Conclusion

CBCT provides the detailed observation of inner tooth anatomy and can be helpful in diagnosis and treatment of abnormal teeth.
